# Prediction of Adsorption and Diffusion of Shale Gas in Composite Pores Consisting of Kaolinite and Kerogen using Molecular Simulation

**DOI:** 10.1021/acs.jpcc.3c00499

**Published:** 2023-05-16

**Authors:** Noura Dawass, Manolis Vasileiadis, Loukas D. Peristeras, Konstantinos D. Papavasileiou, Ioannis G. Economou

**Affiliations:** † Chemical Engineering Program, Texas A&M University at Qatar, P.O. Box 23874, Education City, Doha, Qatar; ‡ Molecular Thermodynamics and Modeling of Materials Laboratory, Institute of Nanoscience and Nanotechnology, National Center for Scientific Research “Demokritos”, GR-15310 Aghia Paraskevi, Attikis, Greece

## Abstract

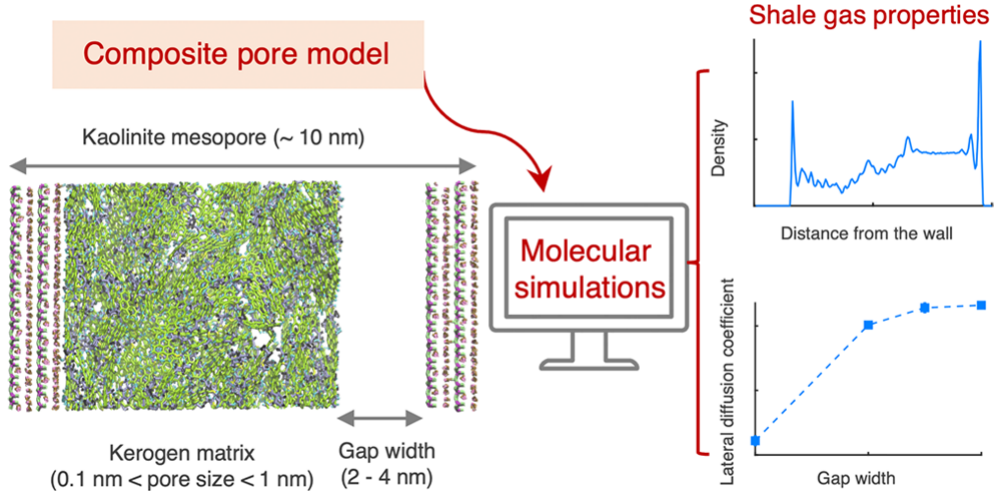

Natural gas production from shale formations is one of the most recent and fast growing developments in the oil and gas industry. The accurate prediction of the adsorption and transport of shale gas is essential for estimating shale gas production capacity and improving existing extractions. To realistically represent heterogeneous shale formations, a composite pore model was built from a kaolinite slit mesopore hosting a kerogen matrix. Moreover, empty slabs (2, 3, and 4 nm) were added between the kerogen matrix and siloxane surface of kaolinite. Using Grand–Canonical Monte Carlo (GCMC) and molecular dynamics (MD) simulations, the adsorption and diffusion of pure methane, pure ethane, and a shale gas mixture were computed at various high pressures (100, 150, and 250 atm) and temperature of 298.15 K. The addition of an inner slit pore was found to significantly increase the excess adsorption of methane, as a pure component and in the shale gas mixture. The saturation of the composite pore with methane was observed to be at a higher pressure compared to ethane. The excess adsorption of carbon dioxide was not largely affected by pressure, and the local number density profile showed its strong affinity to kerogen micropores and the hydroxylated gibbsite surface under all conditions and pore widths. Lateral diffusion coefficients were found to increase with increasing the width of the empty slab inside the composite pore. Statistical errors of diffusion coefficients were found to be large for the case of shale gas components present at low composition. A larger composite pore configuration was created to investigate the diffusion of methane in different regions of the composite pore. The calculated diffusion coefficients and mean residence times were found to be indicative of the different adsorption mechanisms occurring inside the pore.

## 1 Introduction

As the world’s energy demand is rising, unconventional energy resources are becoming increasingly important, especially the ones that provide clean and low-carbon-footprint energy.^1,2^ An example of such an energy source is unconventional natural gas, including shale gas, coalbed methane, and gas hydrates.^3−6^ Shale gas is already a significant player in the world’s energy scene. In 2019, approximately 60% of the U.S. gas production was accounted for by shale gas;^7^ by 2022, that percentage rose to 80%.^8^ While advanced technologies such as horizontal drilling and hydraulic fracturing have boosted the production of shale gas and overcome the low permeability of shale formations, extracting shale gas is still considered challenging.^9^ This is partially due to the difficulty of accurately predicting the properties of shale gas, which is caused by the structural and chemical heterogeneity of shale formations, where pores of micro- (less than 2 nm), meso- (2–50 nm), and macrosizes (larger than 50 nm) are present. Moreover, shale matrices are made up of inorganic (e.g., clay minerals, quartz, and calcite) and organic matter. The solid organic matter, which is insoluble in water and typical organic solvents, is called kerogen.^10,11^

This heterogeneity results in three possible locations for the gas in shale formations: (1) free gas in large pores and fractures, where mass transport is due to viscous forces; (2) gas adsorbed on the shale matrix; and (3) gas dissolved in kerogen.^8^ For the latter two cases, confinement effects are strong, and transport in small pores (meso and microsize) cannot be described by continuum fluid theories.^11,12^ Experiments reveal that the shale matrix is composed of interconnected, considerably smaller kerogen regions that are hosted in inorganic pores.^13,14^ Kerogen networks enjoy large surface areas, resulting in substantial amounts of adsorbed fluids.^11,15^ In fact, gas adsorbed in shale matrices accounts for 20–80% of the total gas in place.^12^ Consequently, understanding the mechanisms governing gas adsorption and transport in the variety of environments found in shale formations is essential for designing and optimizing shale gas extraction processes. Knowledge of shale gas adsorption relates directly to the production capacity of a shale play, and understanding transport mechanisms provides insights into long-term production behavior.^16,17^

Substantial research efforts have been motivated by the significance of shale gas and the complexity of its extraction.^4,9,12,14,17−20^ Different experimental techniques were effective in identifying field compositions, describing porosity, and measuring various macroscopic properties, including adsorption isotherms.^20−23^ However, with the current experimental techniques, it is still challenging to visualize the transport of fluids in meso- and micropores and reveal the underlying adsorption/desorption mechanisms.^11^ In this respect, molecular modeling is a powerful tool that can address fluid behavior and account for confinement effects. Generally, the models underlying the molecular simulation studies related to shale gas can be classified into mesoscale and atomistic. Mesoscale approaches, such as lattice Boltzmann (LB) and kinetic Monte Carlo (KMC), have been mostly used to predict fluid transport and flow in meso- and macropores.^24−28^ The advantage of employing these methods is that heterogeneous shale formations (with different porosity and permeability etc.) can be modeled without significant computational costs.^26,28^ The reader is referred to the corresponding literature for applications of mesoscale methods to the problem of shale gas.^26,28−30^ It should be noted that the performance of such methods depends on adequately describing the interactions present (i.e., fluid–solid and fluid–fluid interactions)^30^ and addressing how properties change with the size of pores.^27^ Given the small scale of the pores occurring in shale formations, atomistic molecular simulations can aid in developing mesoscale models. In the work of Apostolopoulou et al.,^27^ the structure and transport properties of methane in micropores were predicted using molecular dynamics (MD) simulations. These results were used as input to a stochastic KMC model that is capable of reproducing molecular simulations’ results with reasonable accuracy and estimating transport in larger pores (up to ∼60 nm).^27^

Molecular simulation can provide an understanding of how fluid properties change when they are confined in different media. Wang et al.^12^ summarized the various models that have been used to describe inorganic minerals and organic matter when simulating gas adsorption in micropores. The authors have also presented a literature review on the adsorption capacity and mechanisms of inorganic minerals and kerogen. In the study of Wang et al.,^9^ a review of the molecular simulation methods for gas adsorption and diffusion in a shale matrix was presented. To simplify the problem of predicting the properties of natural gas trapped in the shale deposits, the majority of simulation studies at the atomistic level have focused on studying a single type of confined medium consisting of either inorganic material (e.g., montmorillonite, kaolinite, illite) or organic matter (i.e., kerogen).^12^ Approaches using composite models made of organic and inorganic matter to study the adsorption and diffusion of shale gas are very scarce. Examples of such composite models were presented in previous publications.^31−34^ In particular, Lee et al.^31^ investigated the role of interfacial and wettability effects on hydrocarbon desorption and long-time recovery during hydraulic fracturing. The heterogeneous nature of shale was represented by simulating fluids confined in a composite membrane consisting of hydrophobic kerogen surfaces, modeled via carbon nanotubes (CNTs), and hydrophilic quartz surfaces. Zhang and Cao^32^ studied the recovery of shale gas using carbon dioxide while including both inorganic and organic components of shale. Specifically, slit pores of montmorillonite filled with layers of methylnaphthalene were used to represent kerogen. The basal spacing between the montmorillonite sheets was varied, and the displacement of methane by carbon dioxide was found to increase as the size of the pore increased. Huang et al.^23^ also used composite pores to study shale gas recovery and carbon dioxide sequestration. Similar to the aforementioned publication, Na-montmorillonite was selected as the inorganic component, while for kerogen, a more refined model was adopted. The topology of the model was based on the Green River type I kerogen, and it was described by means of the CVFF force field. Molecular simulations of shale gas and carbon dioxide in this heterogeneous pore structure were helpful in demonstrating gas recovery mechanisms and identifying the different gas states (i.e., free, adsorbed, and dissolved) during pressure depletion and carbon dioxide sequestration. Recently, Lyu et al.^34^ computed the adsorption and diffusion of methane in montmorillonite–kerogen (type II-A) composite pores at high pressures. Slit pores were formed between a clay surface and a kerogen matrix. Results showed that variations in the density distribution of methane inside the pore corresponded to different surfaces. These findings indicate that using structures composed of inorganic surfaces (e.g., clay) and kerogen presents a more realistic setup that can provide insights into the behavior and properties of shale gas under the combined effect of both materials.

The objective of this study is to investigate the behavior of a shale gas mixture in composite pores composed of a mature type II kerogen layer (organic) embedded between two kaolinite (clay) walls at various distances from the siloxane basal surface. While in reality, shale samples are of far more complex geometry and constitution, studying the behavior of shale gas in the proposed structure is more realistic than examining its behavior in a clay pore or in a kerogen matrix, separately. The paper is organized as follows: In section 2, the methodology for constructing composite pores and simulation details are presented. In section 3, the thermodynamic and transport properties are reported for methane (CH_4_), ethane (C_2_H_6_), and a CH_4_–C_2_H_6_–nitrogen (N_2_)–carbon dioxide (CO_2_) mixture with a composition resembling that of shale gas in different composite pore widths at a standard temperature and three different pressures (100, 150, and 250 atm). The amounts of fluid adsorbed are computed by employing an algorithm of iterative steps of Grand Canonical Monte Carlo (GCMC) and MD simulations. Additionally, MD simulations in the *NVT* ensemble are performed to compute density profiles and diffusion coefficients. Finally, the main findings of this work are summarized in section 4.

## 2 Methods

### 2.1 Force Field

In this work, three gas fluids were studied: pure CH_4_, pure C_2_H_6_, and a shale gas mixture composed of CH_4_, C_2_H_6_, N_2_, and CO_2_ with the composition in mole fractions being equal to 0.85, 0.07, 0.04, and 0.04, respectively. The properties of these systems were examined in kaolinite slit pores hosting a kerogen layer. All of the components were modeled using force fields already validated in our previous publications.^35−37^ Kaolinite was modeled using ClayFF,^38^ which is one of the most widely used force fields for modeling clay minerals found in shales.^9^ For kerogen, the type II-D model was used, proposed by Ungerer et al.^39^ based on experimental data from the Duvernay shale. This model’s molecular formula is C_175_H_102_N_4_O_9_S_2_; its molecular weight is 2468.8 g mol^–1^, and the characteristic size corresponding to the molecule’s flake effective diameter is 2.8 nm.^39^ While in reality, kerogen is a polydisperse multicomponent mixture, this approach assumes a simplified representation where bulk kerogen can be built from a single type of molecular unit of molecular weight below the estimated solubility limit.^40^ The interactions exhibited in the systems were modeled using (a) the General AMBER Force Field (GAFF) with the same parameters as the ones used previously^36,37^ for kerogen and (b) the Transferable Potentials for Phase Equilibria (TraPPE) force field^41,42^ with united atom description and fixed bond lengths for CH_4_, C_2_H_6_, N_2_, and CO_2_ gases. A cutoff distance of 1.2 nm was applied for Lennard–Jones (LJ) and Coulombic interactions, using the Ewald summation method to handle long-range electrostatic interactions.^43^ Finally, the Lorentz-Berthelot mixing rules were employed for LJ interactions between dissimilar sites.^44,45^

### 2.2 Constructing Composite Pores

**Figure 1:**
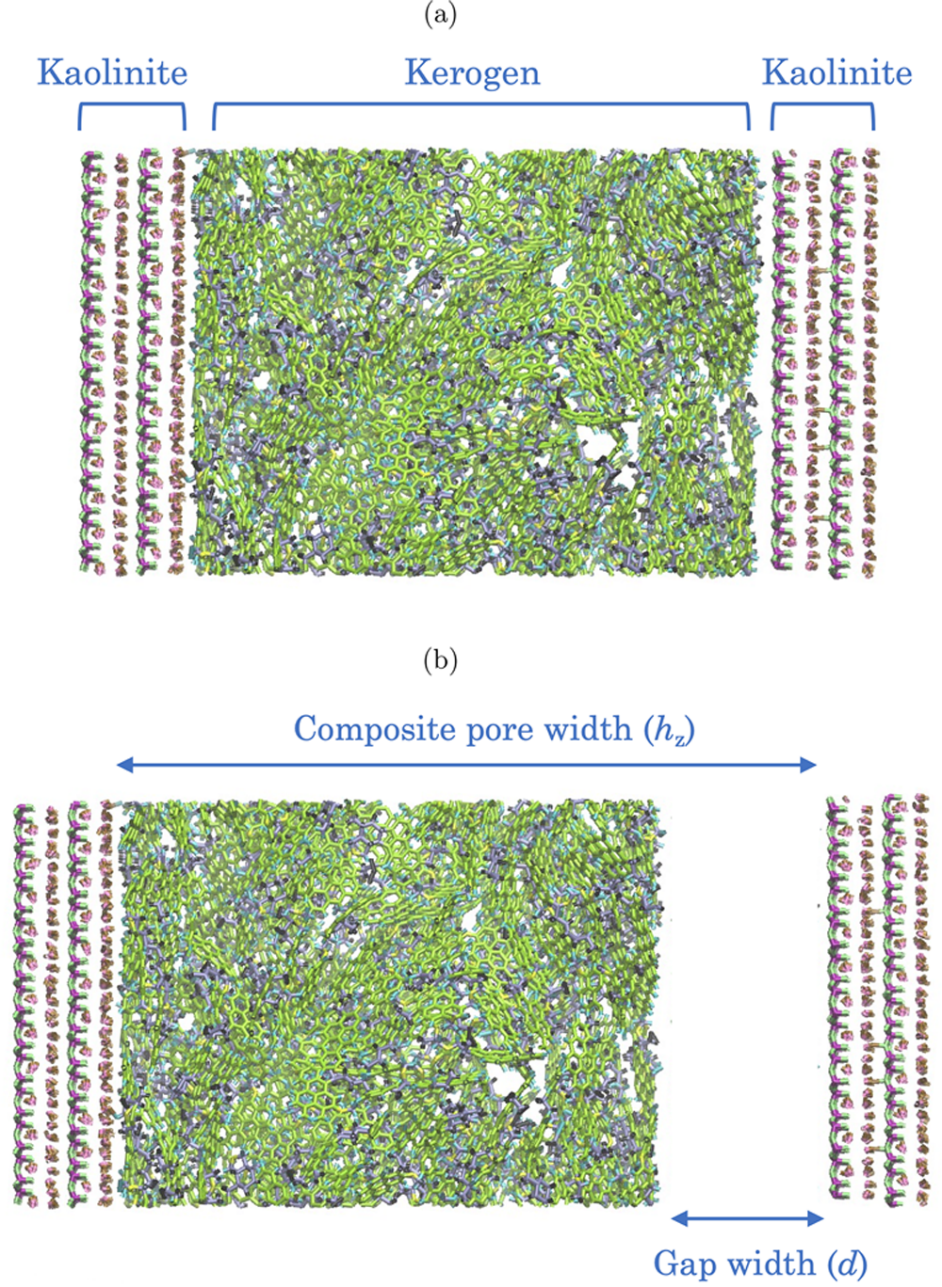
(a) A snapshot of a pore configuration composed of kaolinite mesopores with a kerogen porous matrix. (b) A snapshot of a composite pore made up of four kaolinite layers, kerogen, and an inner mesopore. *h*_*z*_ is the wall to wall distance, normal to kaolinite surfaces, and is used to refer to the total width of the mesopore. *d* is the width of the empty slab between the siloxane surface (right) and kerogen. For all pores constructed in this work, kerogen is placed next to the gibbsite surface (left). Figures were produced using the software package VMD.^77^ In Table 1, values of *h*_*z*_ and *d* are provided for the pores used in this work.

As a first step for creating the composite system, a kaolinite slit mesopore was created by spatially replicating its unit cell (UC) to produce an *n*_*x*_ × *n*_*y*_ × *n*_*z*_ supercell with four inorganic layers along the *z* direction (i.e., *n*_*z*_ = 4) where four siloxane surfaces alternate with four fully hydroxylated gibbsite surfaces. The produced triclinic supercell was transformed to orthorhombic.^46,47^ Then, a gap of *h*_*z*_ nm was introduced between the gibbsite and siloxane basal surfaces, creating a pore large enough to host at low density the kerogen II-D molecules that were randomly inserted into the pore using the Amorphous Builder^48,49^ module in Scienomics MAPS software.^50^ Subsequently, the resulting configuration was brought to equilibrium by performing MD simulations in the *NPT* ensemble, initially at 5 atm and 298.15 K for 2 ns, and then at 1 atm and 298.15 K for 10 ns. Details related to the MD simulations are provided in section 2.3, and a snapshot of the resulting structure is shown in Figure 1a.

Following this procedure, composite pores can be constructed where the kerogen slab resembles as closely as possible the characteristics of its bulk counterpart, and the kaolinite–kerogen interface is free of large cavities and stressed molecular conformations. However, as the system size increases (either by increasing the number of kaolinite UCs or the number of kerogen molecules), adjustments to the construction process are required to avoid the appearance of considerable spatial fluctuations in kerogen mass density and large cavities in the pore. Reducing the initial wall-to-wall distance when creating composite pores assists in producing a larger configuration with a reasonable value for the maximum pore diameter (MPD), comparable to that of bulk kerogen models (i.e., 0.91–1.13 nm).^36^ Moreover, preinserting a number of kerogen molecules with their planes parallel to the kaolinite surfaces helps control the MPD in the resulting configuration. In any case, it appears that confinement has an impact on the kerogen slab’s microporosity, which increases in comparison to a bulk system where no extra porosity has been induced.^36^ This results in a pore size distribution and a larger limiting pore diameter shifted to larger values. Nevertheless, to fully understand the effect of the building strategy on the composite pore’s structural characteristics using II-D-like molecular models requires a thorough investigation that goes beyond the scope of the current study.

Finally, to create the composite pore, gaps of *d* = 2, 3, and 4 nm were introduced in the *z* direction to create an empty slab between the kerogen matrix and the siloxane surface of the kaolinite pore, as shown in Figure 1b. Hereafter, *h*_*z*_ and *d* will refer to the total width of the mesopore (i.e., the distance between the exposed siloxane and gibbsite surfaces) and the width of the empty slab (gap), respectively.

Two composite pores were examined in this work. The first pore, referred to as pore 1, consists of 7 × 6 × 4 kaolinite unit cells and 20 type II-D kerogen molecules. The adsorption and the diffusion coefficients of CH_4_, C_2_H_6_, and shale gas components were calculated using pore 1. The second pore has a larger surface area and was employed to further investigate CH_4_ transport properties in a composite shale formation environment. This pore, referred to as pore 2, is made of 10 × 6 × 4 kaolinite UCs and 55 type II-D kerogen molecules. In Table 1, the dimensions and structural properties of the composite pores used in this work are provided. In the initial low-density configuration, the simulation cell edge along the *z* dimension (*L*_*z*_) was 100.0 and 20.0 nm for pore 1 and pore 2, respectively. Finally, it should be noted that the kaolinite layers’ thickness (roughly 2.95 nm) and the minimum width of the gap introduced (*d* = 2 nm) were much larger than the cutoff distance (*r*_cut_ = 1.2 nm) used for energy and force calculations in order to avoid strong interactions between adjacent interfaces existing in the pores. Details on the porosity characterization and stability of the organic part of the composite pores can be found in section 1 of the Supporting Information (SI).

**Table 1:** Dimensions and Accessible Volume (*V*_acc_) for the Composite Pores Configurations Equilibrated in Step a of the GCMC–*NVT* MD Loading Scheme (see Section 2.3.1)^a^

pore	composition	*d* [nm]	*h*_*z*_ [nm]	*V*_acc_ [nm^3^]
pore 1	7 × 6 × 4 kaolinite UCs (3.63, 5.38, *h*_*z*_ + 2.92 nm), 20 kerogen molecules	0	4.21	7.70
		2	6.21	43.90
		3	7.21	83.12
		4	8.21	63.41
pore 2	10 × 6 × 4 kaolinite UCs (5.20, 5.39, *h*_*z*_ + 2.92 nm), 55 kerogen molecules	4	11.26	113.66

a The *V*_acc_ values are computed with the analytical algorithm proposed by Dodd and Theodorou^63^ and used in eq 2 for pores with the same gap value under all conditions.

### 2.3 Simulation Details

#### 2.3.1 Calculating Adsorption

To compute adsorption, a scheme that combines GCMC and MD simulations was used. To load the composite pores with gas, repeating pairs of GCMC and *NVT* MD simulations were employed using Cassandra^51^ and LAMMPS^52^ software packages, respectively. In the GC ensemble, the temperature and volume of the system are fixed, as is the chemical potential (μ) of a bulk gas reservoir under the same conditions. At equilibrium, the chemical potential of the confined gas is equal to the chemical potential of the bulk. For a specific temperature *T* and pressure *P*, the chemical potential of the fluid was computed with Widom’s particle insertion method^53^ using the Towhee code.^54^ The chemical potential values of the gases studied in this work are provided in section 2 of the SI.

During GCMC simulations, a blend of MC moves was applied for the gas molecules with the following probabilities: 33% insertion, 33% deletion, and 34% translation, with the kaolinite and kerogen parts of the pore kept rigid; instead, during the MD simulations, they were considered flexible and allowed to relax. As a result, the micropores in the kerogen slab adjust to the imposed temperature and pressure conditions (i.e., pore’s loading).

The simulation workflow consisted of the following steps:

(a) Initially, an empty slab of width *d* was added, and an MD simulation in the *NVT* ensemble was performed at 298.15 K for 10 ns to equilibrate the unloaded pore.
(b) Using the last configuration of the MD trajectory, 10^6^ GCMC simulation steps were performed keeping the kaolinite and kerogen parts rigid to load the pores at a preset chemical potential value, corresponding to the mechanical pressure *P* (lithostatic) applied to the system.
(c) Starting from the configuration with loading closer to the mean value specified in step b, an MD simulation in the *NVT* ensemble was performed for 0.25 ns with a flexible pore matrix to equilibrate the loaded system.
(d) Steps b and c were repeated until the loaded number of molecules plateaued (roughly after completing 10 ns of MD simulations).

It should be noted that similar procedures where *NPT* MD is used instead of *NVT* MD are employed as working equivalents of Grand Canonical *NPT* Monte Carlo simulations for estimating the swelling properties of materials.^55−57^ Nevertheless, in this study, we model the system as a slit pore of kaolinite filled with kerogen molecules with a gap artificially introduced between the inorganic and organic parts of the pore. Consequently, as is typical in other studies of gas adsorption in slit mesopores, it is assumed that the mechanical stability is imposed by a pressure exerted on the walls as part of the formation hosting the pore, i.e., the lithostatic pressure.

For the MD simulations in the *NVT* ensemble, the integration time step was set to 1 fs, and the temperature was controlled by a Nosé-Hoover thermostat, with a coupling constant of 0.1 ps.^58^

#### 2.3.2 Computing Diffusion Coefficients from Molecular Dynamic Simulations

The diffusion coefficients of the gases under consideration were determined by *NVT* MD simulations performed using the GROMACS^59−61^ engine. For each system, the starting point was the final configuration produced by the iterative GCMC–MD simulations performed. The production runs lasted 250 ns for the gases in pore 1 and 100 ns for the CH_4_ in pore 2. The integration time step was set to 1 fs, and the Nosé-Hoover thermostat with a coupling constant of 0.2 ps was employed to control the temperature.^58^

For a given gas fluid component *i*, the lateral self-diffusion coefficient, *D*_*xy*,*i*_, was computed using the Einstein relation:
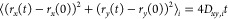
1where *r*_*k*_(*t*) is the *k* component of the molecule’s center of mass at time *t* (*k* in {*x*,*y*}), and the average is calculated over all molecules of component *i* and multiple time origins also referred to as the mean-squared displacement of the molecules on the *xy* plane (MSD_*xy*,*i*_).

The diffusion coefficients obtained from eq 1 reflect the average mobility of molecules in the composite slit pore, which depends on the characteristics of the confinement environment. In order to distinguish the different confinement effects, the mobility of CH_4_ in different regions of the pore is investigated using pore 2, with *d* = 4 nm. The regions were defined based on the obtained density profile of CH_4_. This analysis was carried out for the large pore, where the increased number of CH_4_ molecules results in better statistics of the results obtained in each region, namely, the residence time, *t*_r_, and lateral diffusion coefficients, *D*_*xy*,CH_4__. In the Fickian regime, *D*_*xy*,*i*_ is computed directly from the slope of the MSD_*xy*,*i*_(*t*) curve.^62^ Nevertheless, when Fickian diffusion is not reached, indicative coefficients can be obtained from the slope of the MSD versus time curve at the mean residence time. To compute standard deviations, block averaging with five samples calculated from 10 ns intervals was used.

## 3 Results and Discussion

In this section, we report the properties of pure CH_4_, pure C_2_H_6_, and the mixture representing shale gas. The excess adsorption, the density profiles, and the diffusion coefficients of the gases were calculated from simulations in pore 1. However, their mobility in different regions of the composite pore was investigated using the larger system, i.e., pore 2.

### 3.1 Adsorption

**Figure 2:**
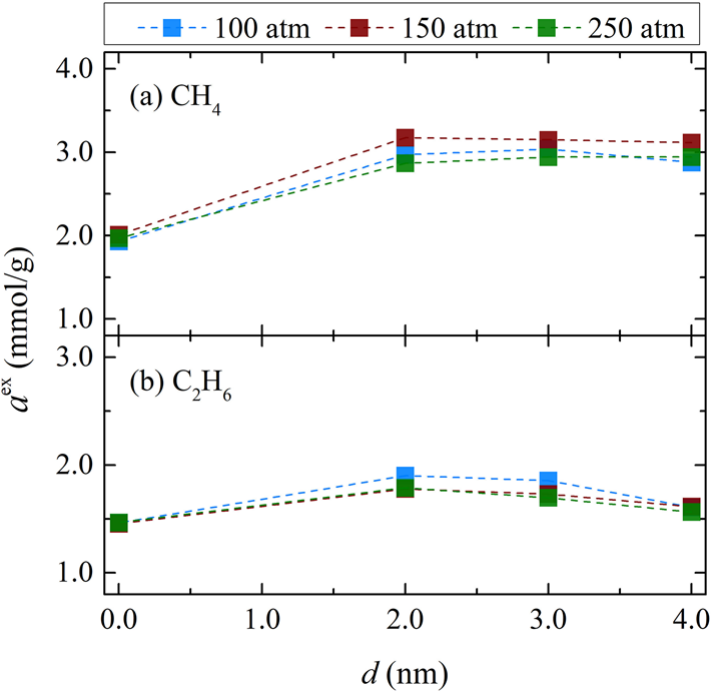
Excess adsorption of (a) pure CH_4_ and (b) pure C_2_H_6_ at 298.15 K and three different pressures as a function of the gap width of composite pore 1. The number of adsorbed molecules is obtained from GC-MD simulations (see section 2.3 for simulation details). Excess adsorption is computed using eq 2. Tabulated values of the computed excess adsorptions along with their standard deviations are presented in section 2 of the SI.

**Figure 3:**
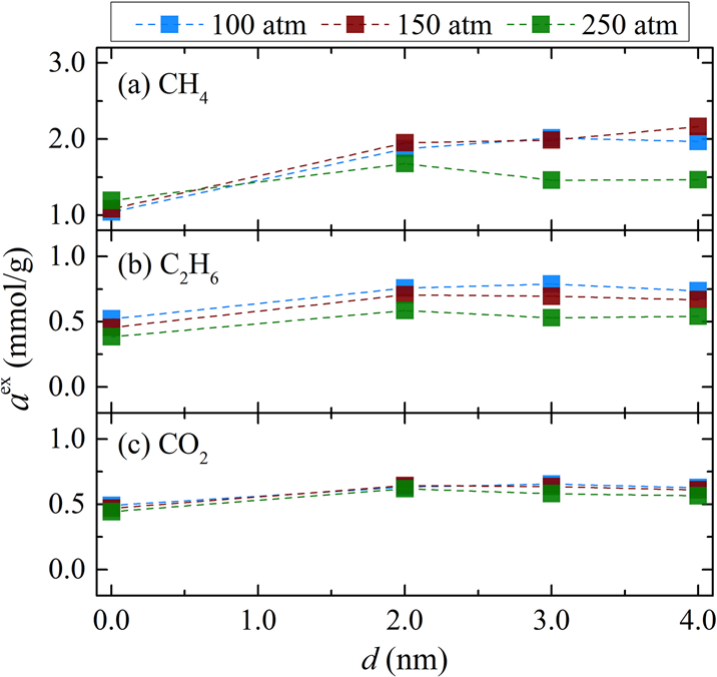
Excess adsorption of the components of the shale gas mixture: (a) CH_4_, (b) C_2_H_6_, and (c) CO_2_ at 298.15 K and the pressures examined, as a function of the gap width of composite pore 1. The number of adsorbed molecules is obtained from GCMC-MD simulations (see section 2.3 for simulation details). Excess adsorption is computed from eq 2. Tabulated values of the computed excess adsorptions along with their standard deviations are presented in section 2 of the SI.

The excess adsorption, *a*^ex^, of pure gases and the shale gas mixture components is presented in Figures 2 and 3, respectively, as a function of the empty slab width *d* for the various pressures studied at *T* = 298.15 K. Expressed as mmol of gas per gram of solid, the *a*^ex^ for each gas is computed from the difference between the amount adsorbed in the pore and the amount that would be present if the gas occupying the pore had a density equal to its bulk density under the same conditions:
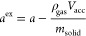
2where *a* is the adsorption computed directly from GCMC-MD simulations, ρ_gas_ is the density of the gas under the simulation’s conditions, and *m*_solid_ is the mass of the solid part of the pore. Gas density was computed from the *NPT* MC simulations performed for the calculation of the chemical potential (see Table S1). The volume of the pore accessible to the gas, *V*_acc_, was calculated using the analytical algorithm proposed by Dodd and Theodorou.^63^ For each pore with a gap value of *d*, the *V*_acc_ values used in eq 2 were determined from the unloaded structure equilibrated in step a of the GCMC–*NVT* MD scheme (see section 2.3.1), independent of the pressure. These values for the composite pores used in this work are reported in Table 1. We should note that if the accessible volume calculated from the *NVT* MD simulation for the estimation of the diffusion coefficient of the gases is used instead, the results would be qualitatively the same. In this scenario, we verified that the excess loading for *d* = 0 nm is not affected, while for *d* > 0 nm, *a*^ex^ is shifted to higher values by practically the same amount (that slightly decreases with increasing pressure). In all cases, no significant changes were observed in the accessible volume over the duration of long MD simulations (see Figure S4 of the SI). The volume, and consequently the width of the organic layer, remains practically unaltered after an initial expansion due to the addition of the gap between the kerogen and the siloxane surface.

Figure 2a shows the excess adsorptions of pure CH_4_ at 298.15 K for various values of *d*; it is demonstrated that the addition of the empty slab results in a significant increase in the excess adsorption. However, as the gap between the kerogen and siloxane surface widens, the increase in CH_4_ uptake is offset by an increase in accessible volume, resulting in a practically constant *a*^ex^ value. In Figure 2a, it is shown that at 298.15 K and for the composite pore 1, the maximum excess adsorption of CH_4_ is reached at a pressure in the vicinity of 150 atm. According to experiments, adsorption isotherms of CH_4_ in shale formations do not follow the Langmuir adsorption model. Instead, the *a*^ex^ of CH_4_ reaches a maximum at a specific pressure, depending on the shale sample, and then decreases.^64^ In Figure 2b, the excess adsorption of pure C_2_H_6_ at 298.15 K is presented for the gap widths examined. It is evident that the dependence of *a*^ex^ on pressure is marginal at higher pressures (namely 150 and 250 atm). At the intermediate gap values, the *a*^ex^ takes higher values at 100 atm, indicating that the corresponding isotherms present a maximum at a pressure less than or equal to 100 atm. Consequently, for pure C_2_H_6_, the pressure corresponding to the maximum excess adsorption is lower than that of pure CH_4_, as shown in Figure 2 and reported by the experimental work of Huang and coworkers.^64^ This behavior was attributed to the smaller molecular size of CH_4_, which allows for its adsorption in smaller pores. At all pressures, the excess adsorption of C_2_H_6_ increases with the addition of the empty slab (i.e., going from *d* = 0 to *d* = 2 nm). However, this increase is small compared to the corresponding one in the case of pure CH_4_. Moreover, by increasing the width of the gap *d* beyond 2 nm, the value of *a*^ex^ clearly decreases, for all pressures examined. Specifically, at *d* = 4 nm, the accessible volume is large enough to counteract the higher uptake of C_2_H_6_, resulting in an excess loading value similar to the value observed in the absence of the gap between the kerogen and siloxane surface.

In Figure 3, the excess adsorption of the shale gas mixture with the aforementioned composition is presented. For all components, the addition of the empty slab results in a typical increase in their excess adsorption observed also for the pure gases. For CH_4_ (see Figure 3a) and C_2_H_6_ (see Figure 3b), the maximum of the isotherm for each *d* value, is located around 150 and 100 atm, respectively, similarly to their pure counterparts. Moreover, it is observed that *a*^ex^ is reduced, compared to their values in the pure systems, for both CH_4_ and C_2_H_6_ by 1 mmol/g approximately. This difference can be accounted for by the adsorption of the other components. For all pressures and gap sizes, the excess adsorption of CH_4_ is at least 2 times larger than that of the excess adsorption of C_2_H_6_ (Figure 3b) and CO_2_ (Figure 3c), which are of similar value, and their dependence on the slab width and pressure follow the same trend. Nonetheless, their excess adsorption values do not seem to vary significantly with pore size compared to CH_4_. The excess adsorption of N_2_ (provided in section 3 of the SI) is the lowest and is close to zero. This could be attributed to the low affinity of N_2_ toward the surfaces of the composite pore. To further investigate the local behavior of the gases adsorbed, density profiles are presented in the following section.

### 3.2 Preferential Distribution

In this section, we present and discuss the number density (ρ_*N*_) profiles of the fluids studied in composite pore 1 with *d* = 4 nm. The calculated density profiles for the remaining systems are briefly discussed here and provided in section 4 of the SI.

**Figure 4:**
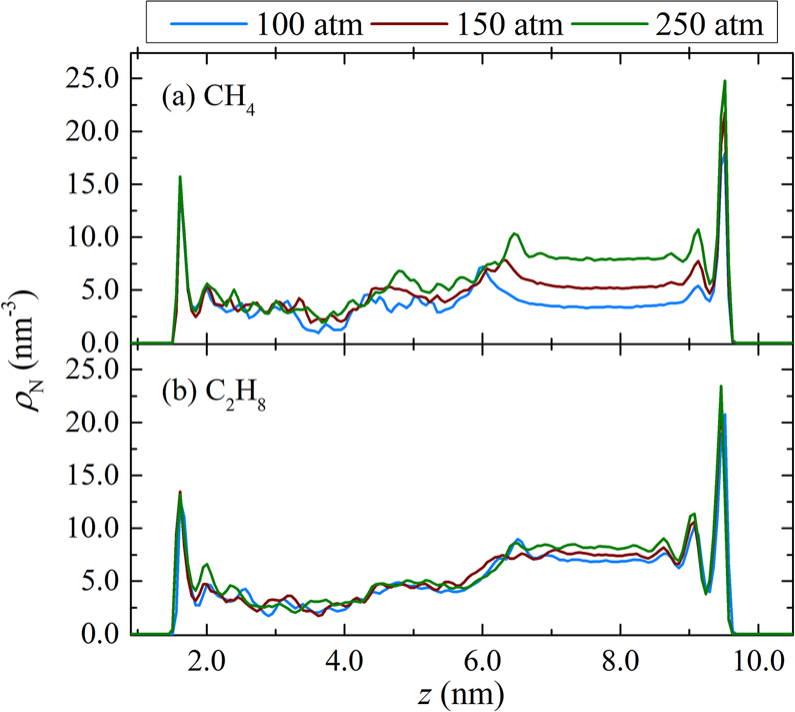
Number density profiles along the axis normal to the kaolinite surfaces (*z* axis) of (a) CH_4_ and (b) C_2_H_6_ in composite pore 1 with *d* = 4 nm at 298.15 K and various pressures.

In Figure 4, the preferential distributions of pure CH_4_ and pure C_2_H_6_ for pore 1 with *d* = 4 nm at 298.15 K and various pressures are shown. For both systems, the number density profiles obtained at all pressures are asymmetrical, with the larger peaks located at a distance of 0.28 nm away from the siloxane surface (higher *z* values), indicative of its strong affinity toward small hydrocarbons.^35^ Generally, the differences in the intensity of the peaks near the two inorganic surfaces increase with the addition of the empty slab (see Figures S5 and S6 in the SI). The siloxane surface (on the right side) is totally accessible to the gas, while the gibbsite surface (on the left side) is slightly more accessible after adding an empty gap as a result of the modest expansion of the organic region. As demonstrated by the reduced intensity of the corresponding peak in the kerogen density profile (Figure S3 in the SI), the empty gap alleviates the packing of kerogen next to the gibbsite surface. For both surfaces, the first adsorption layer, corresponding to the large peak, is followed by a secondary adsorption layer with a smaller height farther away from the surface (approximately 0.4 nm from in the case of the siloxane wall). Moreover, the ρ_N_ value for CH_4_ and C_2_H_6_ in the empty slab region is larger compared to its counterpart in the kerogen-rich region of the composite pore (spanning from 1 up to 6 nm in pore 1). This is observed for all pressure and *d* values examined. Furthermore, the plateau value of the density profile in the empty slab region increases monotonically with pressure.

**Figure 5:**
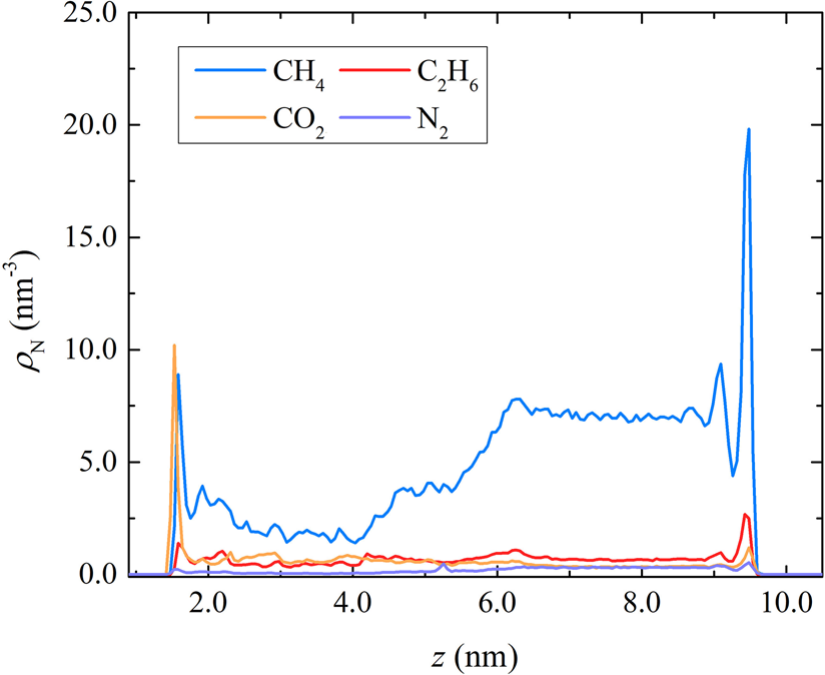
Number density profiles along the axis normal to the kaolinite surfaces (*z* axis) of the components of the shale gas mixture in a composite pore with *d* = 4 nm at 298.15 K and 250 atm.

Figure 5 shows the density profiles for each shale gas mixture component at 298.15 K and 250 atm in composite pore 1 with *d* = 4 nm. The higher value of the CH_4_ number density in the pore is observed next to the siloxane surface. The presence of a significantly smaller number of C_2_H_6_, CO_2_, and N_2_ molecules in the same adsorption layer resulted in the corresponding peaks. The values of ρ_N_ of the components remain almost constant along the region of the gap between the kerogen and siloxane surface, where a plateau is formed extending from 9 nm down to 6 nm. Moving toward the gibbsite surface, at distances less than 6.5 nm (i.e., within the kerogen region), the density starts to decrease for CH_4_ and N_2_ while it increases for CO_2_. The density of C_2_H_6_ remains relatively constant. Finally, a number of distinct peaks in components’ number density are present next to the gibbsite basal surface. Interestingly, the higher peak formed closer to the hydroxylated surface corresponds to CO_2_. With respect to their pure counterparts, the adsorption of CH_4_ and C_2_H_6_ on the gibbsite surface is suppressed. Their adsorption layer, located 0.44 nm away from the siloxane surface, is shifted farther away by 0.17 nm with respect to the pure systems. Consequently, the height of the corresponding peaks was reduced, slightly for CH_4_ and rather considerably for C_2_H_6_. The suppression of CH_4_ adsorption by CO_2_ on kaolinite’s gibbsite surfaces was also reported in the study by Liu et al.^65^ The favorable adsorption of CO_2_ on the gibbsite surface over the other components is also observed at other pressures and pore sizes (see Figure S7 of the SI). The higher adsorption of CO_2_ on the kerogen micropores and the hydroxylated gibbsite is attributed to the presence of Coulomb interactions between the charged CO_2_ molecules and these surfaces.^66^ These results demonstrate the effect of the surface chemistry on adsorption behavior and ultimately shale gas extraction processes. The stronger affinity of gibbsite toward CO_2_ over CH_4_ is relevant to shale gas recovery applications.^67−72^

### 3.3 Transport Properties

The lateral diffusion coefficients of the gas species in the pores under investigation are reported and discussed in this section. To better understand the motion of the molecules in the composite pores, the analysis was further extended to specific regions as identified from the gas components’ density profiles.

**Figure 6:**
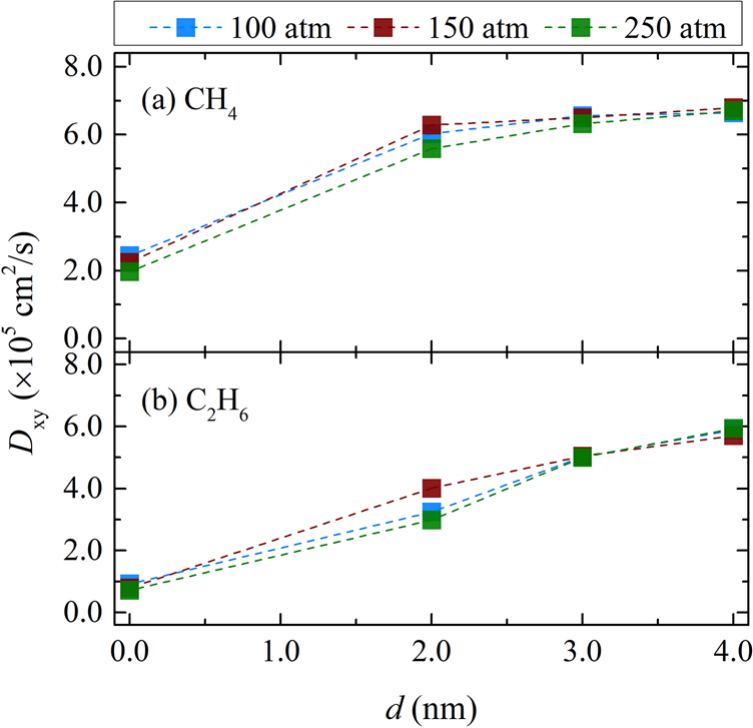
Lateral self-diffusion coefficients (*D*_*xy*_) of (a) pure CH_4_ and (b) pure C_2_H_6_ at 298.15 K and various pressures as a function of the gap width of composite pore 1. Details of the MD simulations are provided in section 2.3. Tabulated values of the computed diffusion coefficients along with their standard deviations are presented in section 3 of the SI.

In Figure 6, diffusion coefficients of pure CH_4_ and C_2_H_6_ at 298.15 K are plotted as a function of the gap width at pressures of 100, 150, and 250 atm. For each gas, the lateral diffusion coefficient increases by expanding the empty slab next to the siloxane surface, and its dependence on *d* for values between 2 and 4 nm is weak for CH_4_ and relatively stronger for ethane. In any case, the values of *D*_*xy*_ at *d* = 4 nm are much lower than their bulk counterparts, as calculated from *NVT* simulations for the system density predicted by the force field in use at the same *T* and *P* conditions (see Table S2 of the SI).

Moreover, for the range of *d* values examined in this work, the self-diffusion coefficients of pure CH_4_ and C_2_H_6_ do not seem to change significantly with pressure. These findings are in agreement with those of earlier simulation studies, where the diffusion coefficient of CH_4_ in inorganic mesopores^73,74^ and kerogen micropores^75^ was found to change marginally for pressures higher than 100 atm. In the case of kaolinite, however, the effect of pressure is significant for pores with basal spacing greater than 6 nm.^73^ Additionally, for a given gap size and pressure, the diffusion coefficient of CH_4_ was found to be always higher than that of C_2_H_6_. At *d* = 0 nm, the diffusion coefficients of pure CH_4_ are roughly double that of pure C_2_H_6_. This ratio was previously reported in the study of Vasileiadis et al.^37^ where the transport of these components was examined in bulk kerogen. As *d* increases, the ratio of the two diffusion coefficients starts to decrease and approaches unity independent of the pressure.

**Figure 7:**
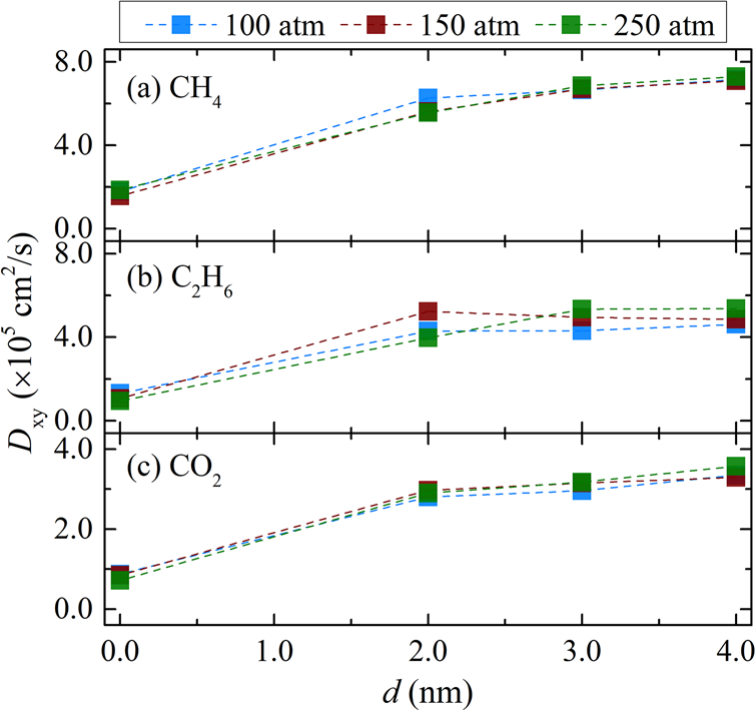
Lateral self-diffusion coefficients (*D*_*xy*_) of the components of the shale gas mixture: (a) CH_4_, (b) C_2_H_6_, and (c) CO_2_ at 298.15 K and various pressures as a function of the gap width of composite pore 1. Details of the MD simulations are provided in section 2.3. Tabulated values of the computed diffusion coefficients along with their standard deviations are presented in section 3 of the SI.

In Figure 7, the lateral diffusion coefficients of the shale gas components are presented. The order of *D*_*xy*_ of the shale gas components is observed to follow their molecular sizes. The diffusion coefficients of CH_4_—the lightest molecule—are the largest, followed by C_2_H_6_ and CO_2_. Compared to pure gases, a similar behavior was observed for the diffusion coefficients of the shale gas mixture components concerning the effect of pressure and the width *d* of the empty slab. Their pressure dependence was minor, and as the slab width exceeds 2 nm, their values are affected slightly for CH_4_ and marginally for the other gases. In this regard, the computed *D*_*xy*_ error bars for C_2_H_6_, CO_2_, and N_2_ were found to be particularly large. This should be attributed to the small number of molecules of these components, resulting in poor statistics in the estimated transport properties. In the case of N_2_, errors on the order of 50% were obtained; thus, the diffusion coefficients of N_2_ are not reported.

#### 3.3.1 Mobility of Methane in Different Regions of Composite Pores

**Figure 8:**
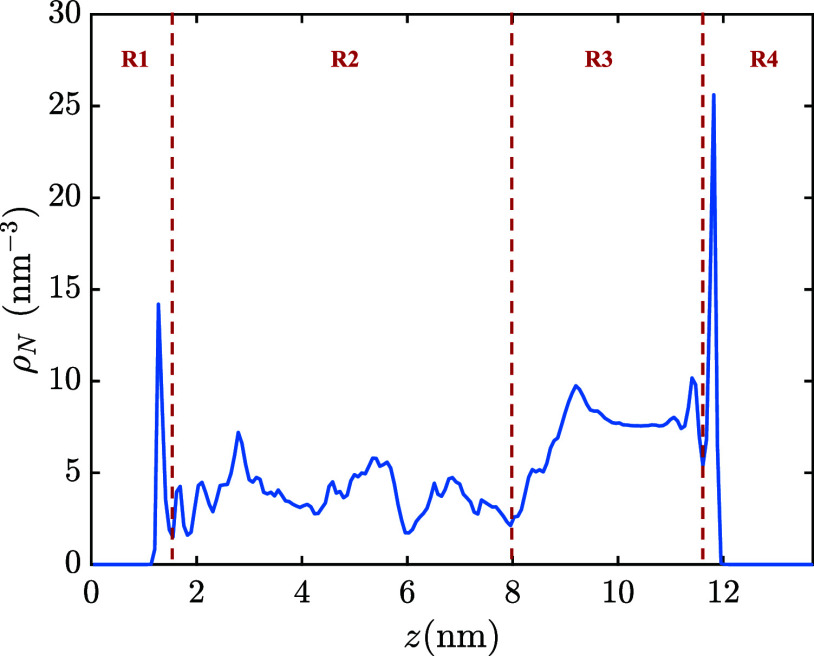
Density profile of CH_4_ along the axis normal to the kaolinite surfaces (*z* axis) inside pore 2. The dashed lines are indicative of the regions defined to analyze the mobility of CH_4_ (see section 3.3.1). R1, adsorption layer next to the gibbsite surface; R2, kerogen-rich part of the pore; R3, the empty slab; and R4, adsorption layers on the siloxane surface.

Finally, the mobility of CH_4_ in different regions of composite pore 2 with *d* = 4 nm was studied. The composite pore was divided into four regions based on the number density profile along the *z* direction (perpendicular to the pore’s surface) shown in Figure 8. Going from lower to higher *z* values, these regions correspond to (a) the adsorption layer next to the gibbsite surface of kaolinite (R1), (b) the kerogen region (R2), (c) the empty slab between the kerogen matrix and the kaolinite wall (R3), and (d) the adsorption layers on the siloxane surface of kaolinite (R4). Trajectories from long MD simulations in the *NVT* ensemble were used to calculate the MSD and the diffusion coefficient of the gases in each region. To improve the estimates, gas molecules immobilized in organic pockets during the simulations were excluded from the analysis. These “trapped” molecules were identified based on their MSD values along the trajectory. They were discovered to be located either directly adjacent to the gibbsite surface or in the kerogen matrix (i.e., either in R1 or R2), and their number was found to be around 2.5% of the total number of CH_4_ molecules adsorbed in the composite pore. Moreover, based on the maximum values of their MSD, the characteristic dimension of the inaccessible pockets is estimated to be 0.4 nm. More details on the criteria used to identify the nonmoving molecules and fit the MSD versus time plots can be found in section 5 of the SI.

In Table 2, the mean residence times τ and the lateral diffusion coefficients *D*_*xy*_ of CH_4_ in different regions of pore 2 are reported. The mean residence time values could be a good indicator of the strength of the developed interactions between CH_4_ and the nearby surface. CH_4_ molecules spend on average 2 ns diffusing in the kerogen matrix (R2), which is characterized by a large surface area. In comparison, the mean residence time of CH_4_ in the empty slab region (R3), which has around half the width of the kerogen region R2, is approximately 15 times less since the molecules are less affected by the confinement. The value of τ in the adsorption layer next to the gibbsite surface (R1) is larger than in R4, although CH_4_ interaction with the siloxane surface is stronger. The presence of the kerogen molecule next to the surface seems to restrict the movement of CH_4_ molecules, forcing them to spend more time on the gibbsite surface compared to the siloxane surface. This is also confirmed by the estimated diffusion coefficients in R1, which is the lowest in the composite pore.

**Table 2:** Mean Residence Times and Diffusion Coefficients for CH_4_ in Different Regions of Composite Pore 2, with *d* = 4 nm^a^

region	mean residence time (τ) [ps]	*D*_*xy*_ × 10^5^ [cm^2^/s]
R1	61.56	0.97 ± 0.14
R2	1989.15	5.30 ± 0.16
R3	131.89	15.82 ± 0.17
R4	37.81	12.46 ± 0.63

a The regions are defined according to the density profile along the *z* axis provided in Figure 8.

The estimated lateral diffusion coefficients of CH_4_ in kerogen were found to be in good agreement with the simulation results of Vasileiadis et al.^37^ for the same kerogen model. Furthermore, the diffusion coefficient in the empty slab region (R3) is approximately 3 times higher than in kerogen and on the same order of magnitude as the diffusion coefficient of bulk CH_4_.^76^

## 4 Conclusions

In summary, the properties of fluids relevant to the process of shale gas extraction were computed in composite pores composed of a kaolinite mesopore hosting a kerogen matrix and a smaller slit pore/empty slab formed between kerogen and the siloxane side of kaolinite. The organic region of the composite pore model presented a relatively higher porosity compared to its bulk counterpart using the II-D kerogen model. This can be attributed to the combined effect of the confinement, the construction procedure, and the specific molecular model used for kerogen and requires further investigation. Atomistic molecular simulations were used to determine the adsorption and diffusion coefficients in various composite pores of pure methane, pure ethane, and a mixture of shale gas. The amount of each fluid adsorbed were computed using a scheme that combines GCMC simulations and MD simulations, which are needed to equilibrate the complex pore structure. Excess adsorptions were calculated at 298.15 K and pressures of 100, 150, and 250 atm, as a function of the width of the empty slab. Adding an empty slab in the composite pore resulted in the increase of absolute and excess adsorption of all gases studied. The pressure at which the pore is saturated was found to be different for CH_4_ and C_2_H_6_. While the highest excess adsorption of C_2_H_6_ was obtained at 100 atm, the maximum excess adsorption of CH_4_ was estimated to be at 150 atm. Density profiles along the axis normal to the kaolinite surfaces (*z* axis) were also computed. From the density profiles of shale gas mixture components, it was concluded that the strong adsorption of CO_2_ can be mainly attributed to the interactions between CO_2_ and the solid surfaces, particularly the hydroxylated gibbsite surface of kaolinite and kerogen. Due to these intermolecular forces, CO_2_ competes for adsorption with other gases, including CH_4_.

Using MD simulations in the *NVT* ensemble, lateral self-diffusion coefficients were computed. CH_4_ was found to have the highest diffusion coefficients compared to the other species. Generally, the order of mobility of molecules (CH_4_ > C_2_H_6_ > CO_2_) was found to follow the order of their molecular size. Larger error bars were observed for the estimation of self-diffusion coefficients compared to error bars from adsorption calculations. Hence, to study the diffusion of CH_4_ in different parts of the composite pore, a larger system was constructed and utilized. The diffusion coefficient in kerogen was found to be much slower than that in the empty slab region, where the diffusion coefficient is on the same order as the bulk diffusion. The kerogen slab of the composite pores was found to be stable during the simulations independent of the pressure and the size of the gap added between the organic and inorganic regions.

## Supporting Information Available

Structural properties of the composite pore and stability analysis of the organic region; the densities and chemical potentials of the gases studied in the bulk phase; the tabulated results for excess adsorption and self-diffusion coefficients with their standard deviations; the density profiles of all gases in the pores examined; additional details related to studying the mobility of methane in different regions (PDF)

## Dedication

This paper is dedicated to Professor Pablo G. Debenedetti, a pioneer in the study of condensed matter systems using statistical mechanics, molecular thermodynamics and molecular simulation.
