# Supplementary material for: Prediction of Adsorption and Diffusion of Shale Gas in Composite Pores Consisting of Kaolinite and Kerogen using Molecular Simulation
Source: J Phys Chem C Nanomater Interfaces. 2023 May 16;127(20):9452–62. doi: 10.1021/acs.jpcc.3c00499 (PMC10863031; doi:10.1021/acs.jpcc.3c00499)
Supplement: Supplementary file 1 — jp3c00499_si_001.pdf [file jp3c00499_si_001.pdf]

Supporting Information: Prediction of Adsorption and Diffusion of Shale Gas in Composite Pores Consisting of Kaolinite and Kerogen using Molecular Simulation

Noura Dawass,† Manolis Vasileiadis,‡ Loukas D. Peristeras,*,‡ Konstantinos D. Papavasileiou,‡ and Ioannis G. Economou*,†

†*Chemical Engineering Program, Texas A&M University at Qatar, Education City, Doha PO Box 23874, Qatar*

‡*Molecular Thermodynamics and Modeling of Materials Laboratory, Institute of Nanoscience and Nanotechnology, National Center for Scientific Research "Demokritos", GR-15310 Aghia Paraskevi, Attikis, Greece*

E-mail: l.peristeras@inn.demokritos.gr; ioannis.economou@qatar.tamu.edu

1 Structural properties and stability of the organic region

The method for creating the hybrid pores' configurations examined in this study, as described in Section 2.2 of the main manuscript, results in microporous organic slabs with enhanced porosity in comparison to their bulk counterparts. The density of the organic region in the composite pores examined in the study is estimated to range between 1.05 and 1.10 g/cm^3 at 298.15K and 1 atm. The pore size distributions (PSD) for pore 1 with $d = 0$ nm are presented in Figure S1a and Figure S1b for 100 atm and 250 atm, respectively. They have been calculated by examining 100 configurations distributed evenly along the NVT MD trajectories for estimating the diffusion of CH_4. Each frame was analysed using Poreblazer software[1] and omitting the gas molecules. Additionally, the PSD at the start, middle, and end of the simulation were obtained by grouping and analyzing frames 1–30, 35–65, and 70–100, respectively. This provides access to the time evolution of the distributions and therefore can be used as a measure of the stability of the pore. The increase in pressure slightly limits the extent of the distribution to higher values and reduces the fluctuation of the pores' size observed during the simulation. The maximum pore diameter (MPD) also reduces from 1.10±0.07 nm at 100 atm to 1.03±0.03 nm at 250 atm. Visual inspection of the configurations reveals that the cavities are evenly distributed across the slab, with no preference given to their proximity to the clay surfaces. Moreover, the mean limiting pore diameter (LPD) reduces from 0.57±0.03 nm at 100 atm to 0.51±0.02 nm at 250 atm. Nonetheless, these values are much higher than their counterparts in the bulk estimated using the same kerogen model without inducing extra porosity,[2] which barely exceeds 0.3 nm. It is worth noticing that the structure of the confined kerogen region resembles the bulk counterparts created by using LJ particles with diameters higher than 3.0 nm[2] in order to increase the available accessible volume.

Adding a gap of 3 nm between the siloxane surface and the kerogen (see Figure S1c and Figure S1d for 100 and 250 atm, respectively), has a minimal impact on the PSD and the stability of the organic slab that seems not to depend on the pressure. The microporosity

is maintained while the time evolution of the distribution suggest that the organic slab is stable with no significant changes in the pore network spanning the kerogen region.

The same behavior is observed for pore 2. Figure S2 illustrates the PSD for pore 2 with $d = 4$ nm where it is evident that the microporosity of the structure does not change during the NVT simulations.

In Figure S3 the number density profile along the direction perpendicular to pore surfaces is presented for pore 2 at $d = 0$ nm and $d = 4$ nm. In the presence of the gap, the organic slab expands towards the empty space. The peak next to gibbsite surface ($z \approx 1.8$ nm) becomes less intense and the kerogen molecule locate near the exposed surface of the organic slab partially repositioned to occupy the empty space. The shape of the created surface is rough and fluctuates during the simulation together with the dividing surface defined by the density profile that is located at approximately 6.2 nm.

Finally, the methane based accessible surface area (S_{acc}) and accessible volume (V_{acc}) of the systems do not change significantly over the course of a 100 ns MD run as shown in Figure S4 for pore 1 with $d = 4$ nm loaded with methane at 250 atm. Note that gas molecules where not accounted for in the calculations.

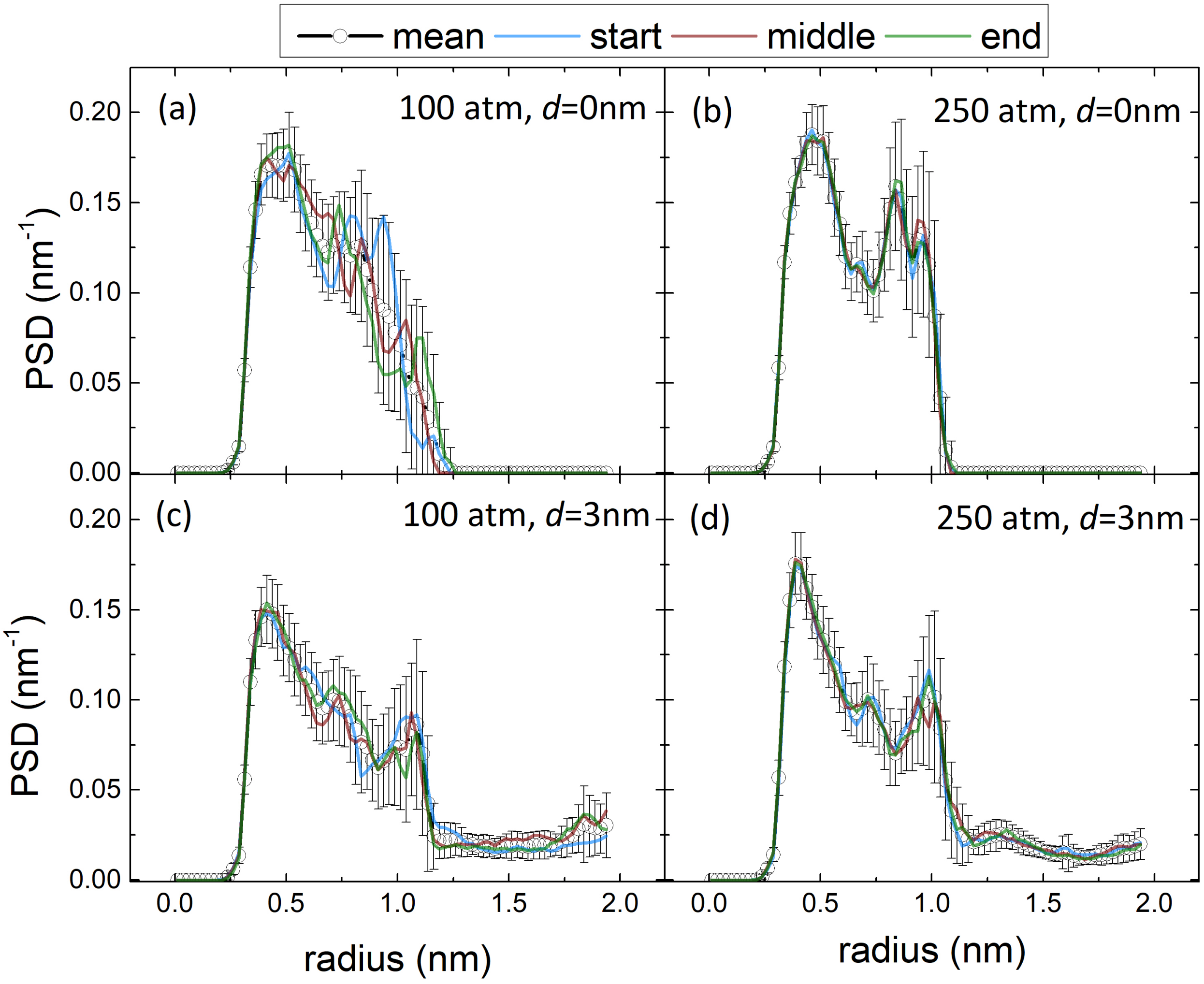

Figure S1: Mean PSD and their time evolution for pore 1 with (a) $d = 0$ nm at 100 atm, (b) $d = 0$ nm at 250 atm, (c) $d = 3$ nm at 100 atm, and (d) $d = 3$ nm at 250 atm, calculated from the NPT MD simulations for estimating methane's diffution in the pore at 298.15 K.

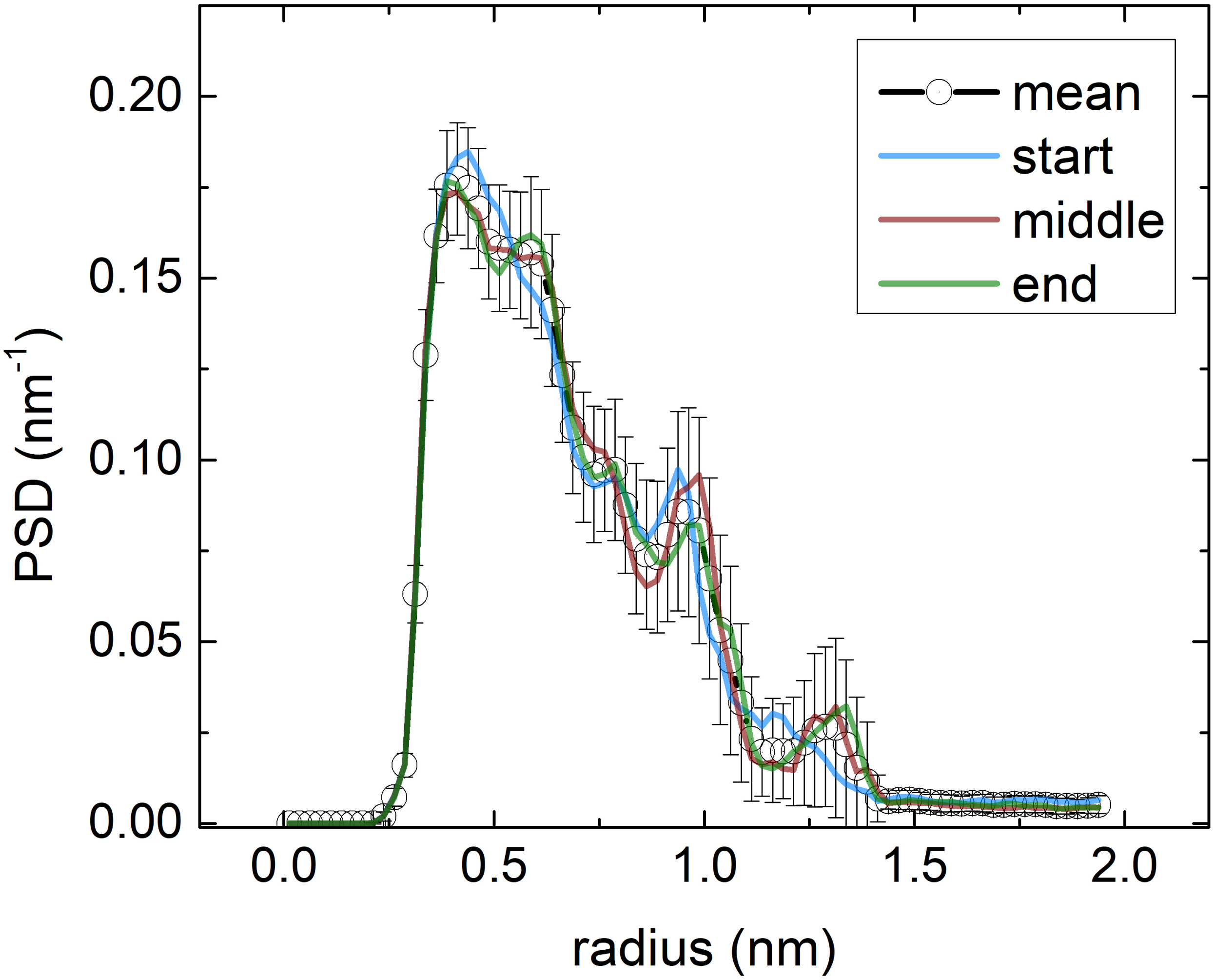

Figure S2: The mean PSD and its time evolution for pore 2 with $d = 4$ nm at 100 atm and 298.15 K, calculated from the NPT MD simulations for estimating methane's diffution in the pore.

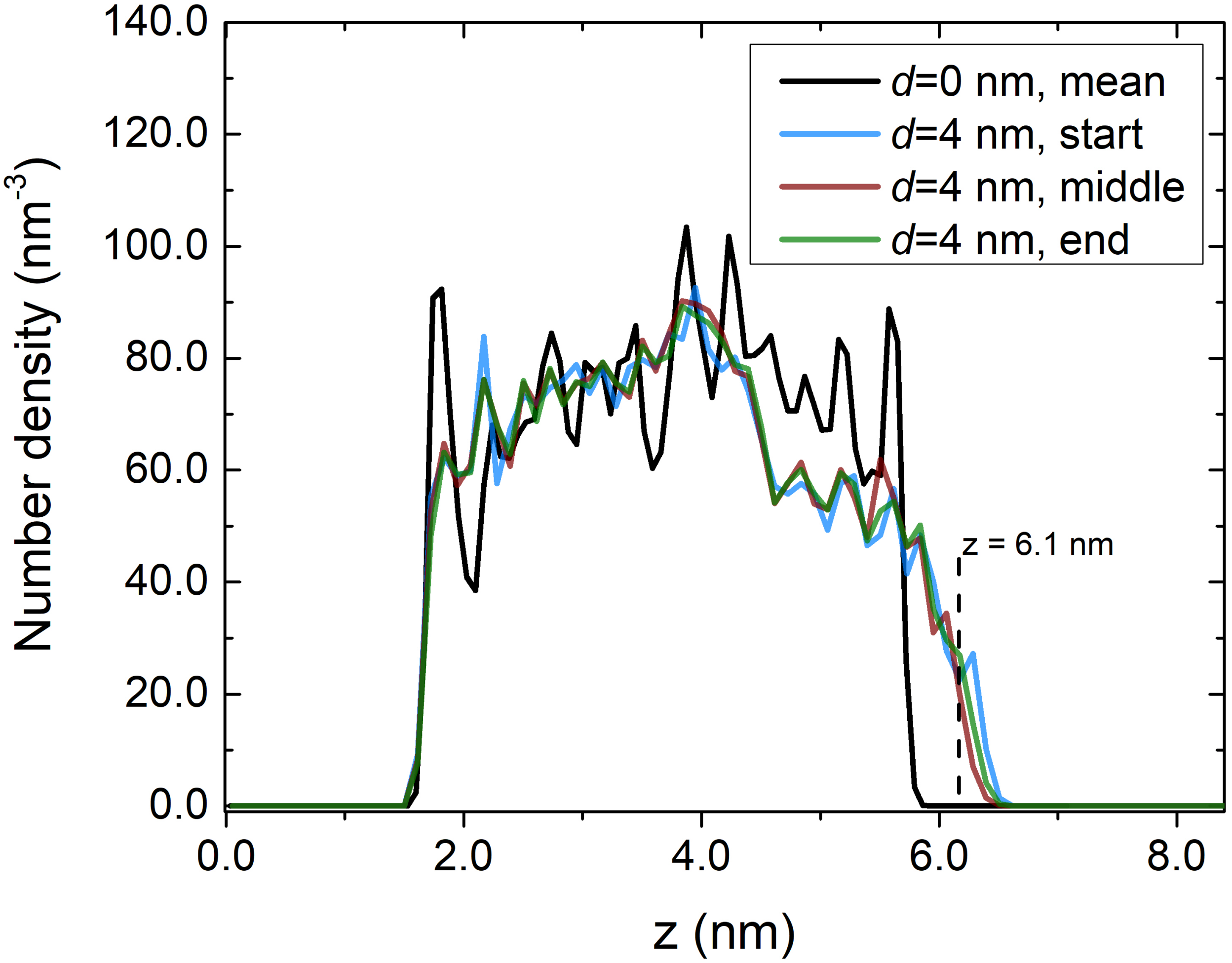

Figure S3: Density profiles along the axis normal to the kaolinite surfaces (z–axis) of kerogen molecules in pore 1 at 250 atm and 298.15 K. The mean density profile is presented for d = 0 nm while its time evolution is presented for d = 4 nm. The former remains practically unchanged during the MD simulations.

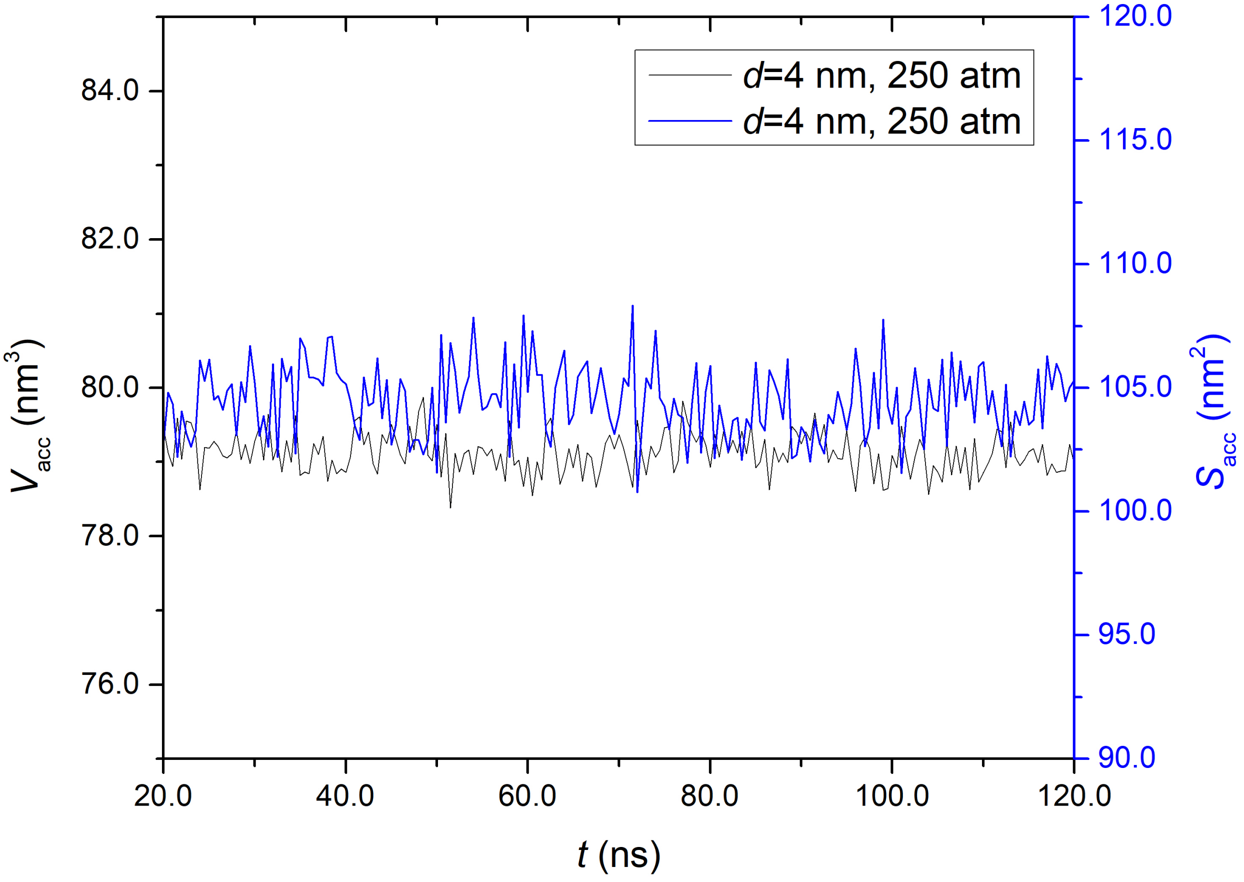

Figure S4: Accessible surface area (S_{acc}) and accessible volume (V_{acc}) of pore 1 with $d = 4$ nm during a MD simulation in the NVT ensemble.

2 Bulk gas properties

Table S1: Bulk densities and chemical potentials of pure CH_4, pure C_2H_6 and the shale gas mixture (with the molar composition: 0.85 CH_4, 0.07 C_2H_6, 0.04 N_2, 0.04 CO_2) at 298.15 K and the pressures studied in this work.

Property	ρ [mol/L]			μ [KJ/mol]		
P [atm]	100	150	250	100	150	250
Pure CH_4	4.86	7.50	11.88	-25.52	-24.67	-23.64
Pure C_2H_6	12.87	13.59	14.55	-29.74	-29.36	-28.72
Shale gas						
CH_4	4.23	6.70	10.58	-25.93	-25.09	-24.05
C_2H_6	0.35	0.55	0.87	-35.40	-35.04	-34.57
CO_2	0.20	0.31	0.50	-37.73	-37.26	-36.68
N_2	0.20	0.31	0.50	-34.86	-33.91	-32.31

Table S2: Self–diffusion coefficients ($D \times 10^5$ cm^2/s) of pure CH_4 and pure C_2H_6 in the bulk phase at $T = 298.15$ K at 100 and 250 atm. MD simulations in the NVT ensemble were used to compute the diffusion coefficients, with the gases modeled using the TraPPE force field.

P (atm)	$D \times 10^5$ (cm^2/s)	
	CH_4	C_2H_6
100	33.88 ± 0.46	15.87 ± 0.09
250	18.30 ± 0.03	13.90 ± 0.09

3 Excess adsorption and lateral diffusion coefficient calculations

Table S3: Excess adsorption (a^{ax}) in mmol of gas per g of solid of pure CH_4 in pore 1 at $T = 298.15$ K and various pressures.

d (nm)	P (atm)		
	100	150	250
0	1.930 ± 0.016	2.009 ± 0.008	1.969 ± 0.009
2	2.969 ± 0.053	3.175 ± 0.064	1.969 ± 0.009
3	3.037 ± 0.020	3.149 ± 0.035	2.943 ± 0.019
4	2.878 ± 0.032	3.116 ± 0.051	2.942 ± 0.022

Table S4: Excess adsorption (a^{ax}) in mmol of gas per g of solid of pure C_2H_6 in pore 1 at $T = 298.15$ K and various pressures.

d (nm)	P (atm)		
	100	150	250
0	1.462 ± 0.012	1.454 ± 0.029	1.466 ± 0.013
2	1.900 ± 0.059	1.777 ± 0.018	1.788 ± 0.019
3	1.855 ± 0.020	1.729 ± 0.024	1.695 ± 0.024
4	1.607 ± 0.051	1.612 ± 0.045	1.695 ± 0.024

Table S5: Excess adsorption (a^{ax}) in mmol of gas per g of solid of the shale gas components in pore 1 at $T = 298.15$ K and various pressures.

d (nm)	P (atm)		
	100	150	250
		CH_4	
0	1.041 ± 0.009	1.081 ± 0.016	1.193 ± 0.039
2	1.873 ± 0.088	1.952 ± 0.089	1.678 ± 0.066
3	2.014 ± 0.035	1.986 ± 0.061	1.460 ± 0.150
4	1.969 ± 0.136	2.164 ± 0.103	1.465 ± 0.086
		C_2H_6	
0	0.521 ± 0.028	0.456 ± 0.032	0.384 ± 0.023
2	0.758 ± 0.038	0.705 ± 0.068	0.585 ± 0.027
3	0.788 ± 0.021	0.695 ± 0.019	0.530 ± 0.050
4	0.736 ± 0.048	0.668 ± 0.022	0.541 ± 0.018
		CO_2	
0	0.491 ± 0.030	0.469 ± 0.023	0.443 ± 0.023
2	0.632 ± 0.047	0.644 ± 0.034	0.619 ± 0.027
3	0.655 ± 0.049	0.636 ± 0.052	0.581 ± 0.050
4	0.627 ± 0.037	0.610 ± 0.088	0.565 ± 0.018
		N_2	
0	0.016 ± 0.000	0.017 ± 0.004	0.024 ± 0.012
2	0.039 ± 0.016	0.005 ± 0.012	0.015 ± 0.019
3	0.029 ± 0.006	0.028 ± 0.009	0.018 ± 0.020
4	0.028 ± 0.012	0.026 ± 0.010	0.016 ± 0.021

Table S6: Lateral diffusion coefficients ($D_{xy} \times 10^5$ cm^2/s) of pure CH_4 in pore 1 at T = 298.15 K and various pressures.

d (nm)	P (atm)		
	100	150	250
0	2.440 ± 0.147	2.243 ± 0.094	1.967 ± 0.232
2	6.028 ± 0.092	6.284 ± 0.236	5.587 ± 0.187
3	6.562 ± 0.147	6.498 ± 0.149	6.321 ± 0.193
4	6.641 ± 0.026	6.793 ± 0.048	6.700 ± 0.131

Table S7: Lateral diffusion coefficients (D_{xy} x 10^5 cm^2/s) of pure C_2H_6 in pore 1 at T = 298.15 K and various pressures.

d (nm)	P (atm)		
	100	150	250
0	0.920 ± 0.055	0.795 ± 0.048	0.719 ± 0.028
2	3.243 ± 0.073	4.009 ± 0.298	2.981 ± 0.030
3	5.017 ± 0.068	5.044 ± 0.045	5.002 ± 0.022
4	5.890 ± 0.103	5.700 ± 0.068	5.936 ± 0.040

Table S8: Lateral diffusion coefficients (D_{xy} x 10^5 cm^2/s) of the shale gas components in pore 1 at T = 298.15 K and various pressures.

d (nm)	P (atm)		
	100	150	250
	CH_4		
0	1.783 ± 0.062	1.567 ± 0.034	1.854 ± 0.172
2	6.263 ± 0.230	5.612 ± 0.457	5.557 ± 0.160
3	6.644 ± 0.122	6.696 ± 0.228	6.854 ± 0.607
4	7.157 ± 0.282	7.086 ± 0.437	7.281 ± 0.264
	C_2H_6		
0	1.308 ± 0.201	1.065 ± 0.114	0.942 ± 0.086
2	4.294 ± 0.246	5.235 ± 0.355	3.970 ± 0.199
3	4.308 ± 0.255	4.955 ± 0.227	5.341 ± 0.813
4	4.614 ± 0.390	4.857 ± 0.266	5.372 ± 0.805
	CO_2		
0	0.880 ± 0.106	0.850 ± 0.027	0.716 ± 0.072
2	2.805 ± 0.305	2.974 ± 0.149	2.904 ± 0.162
3	2.963 ± 0.347	3.152 ± 0.274	3.178 ± 0.537
4	3.352 ± 0.066	3.294 ± 0.435	3.577 ± 0.367

4 Density profiles

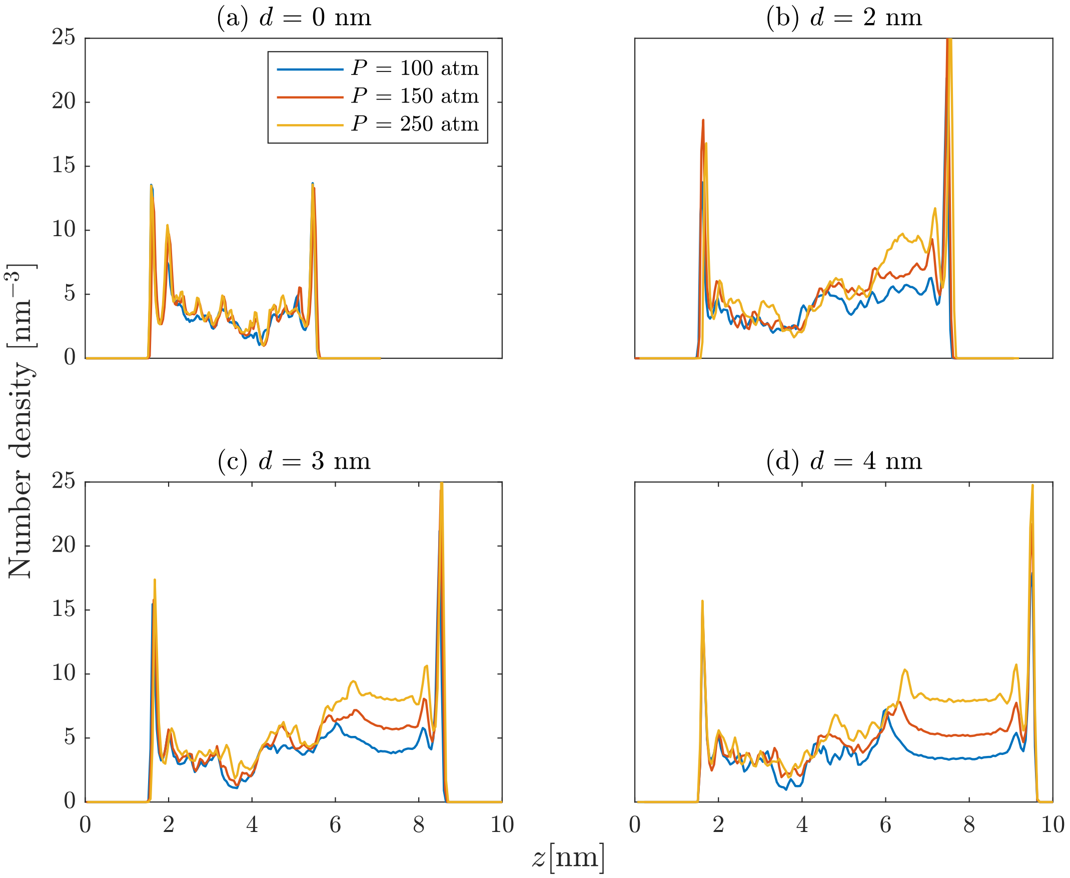

Figure S5: Density profiles along the axis normal to the kaolinite surfaces (z–axis) of pure CH_4 in composite pore 1, at 298.15 K, and various pressures.

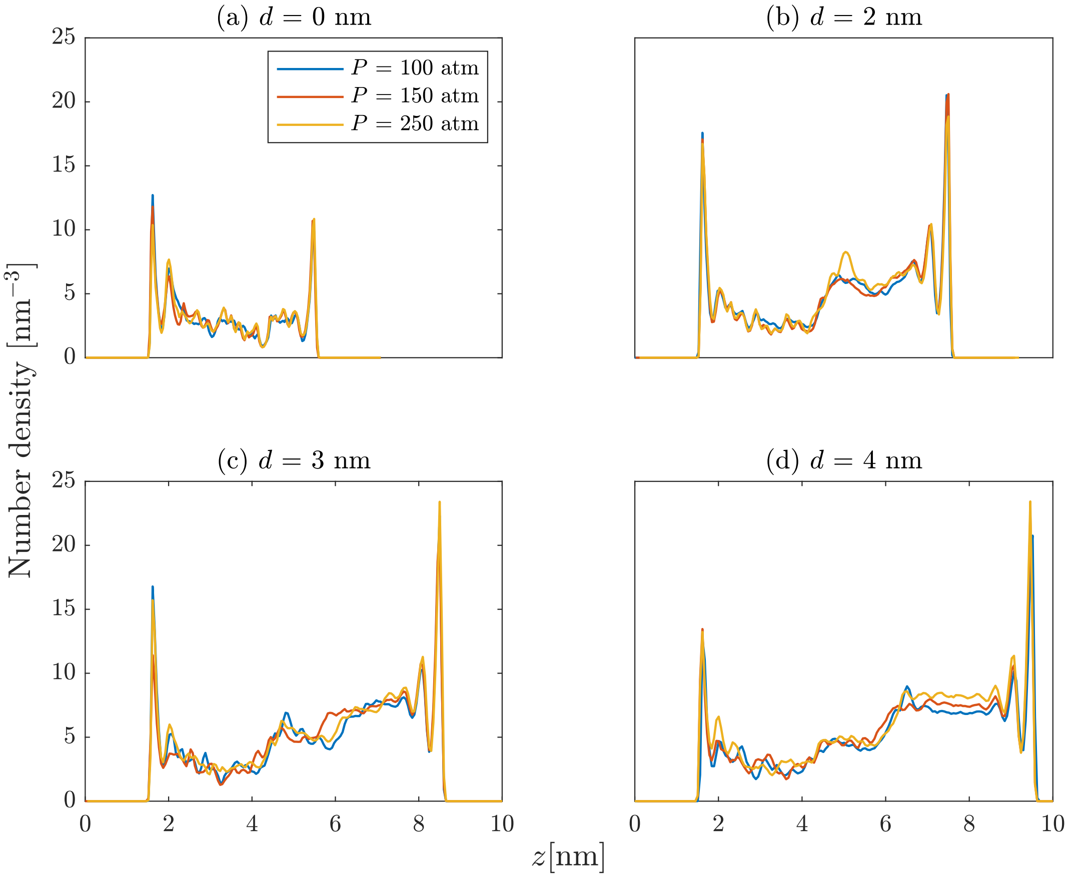

Figure S6: Density profiles along the axis normal to the kaolinite surfaces (z–axis) of pure C_2H_6 in composite pore 1, at 298.15 K and various pressures.

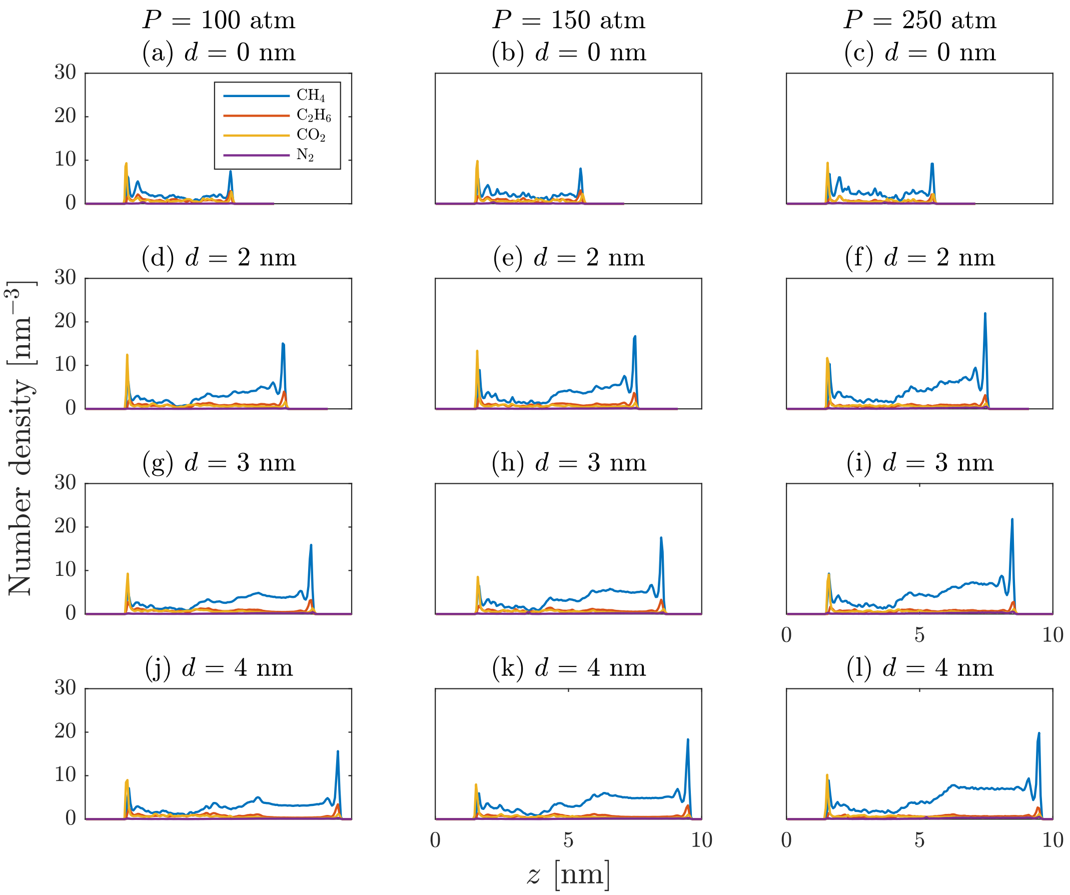

Figure S7: Density profiles along the axis normal to the kaolinite surfaces (z–axis) of the shale gas components in composite pore 1, at 298.15 K, and various pressures.

5 Mobility analysis details

5.1 Identifying trapped molecules

Due to the heterogeneous nature of the composite pores studied in this work, in terms of pore sizes (micro and meso) and adsorption surfaces (kaolinite and kerogen), the variance in the transport rates are expected to be large, compared to that of a homogeneous fluid. For instance, from examining the MSDs and diffusion coefficients of individual molecules, it was found that a number of molecules have very low MSDs and diffusion coefficients near zero. It is beneficial to identify the number of so–called "trapped molecules" and eliminate them from the mobility study of CH_4 in different regions of the composite pore. To identify these trapped molecules, the following steps were carried out:

1. The distribution of MSDs at the final time step of the simulation was plotted as shown in Figure S8a.

2. Based on the distribution and the first peaks formed, which correspond to the entrapment of molecules in small pockets with a diameter of around 4 nm, a cutoff MSD value was used to determine slow molecules ($MSD_{cutoff} = 10$ nm^2).

3. To ensure that these slow molecules were not moving at previous time steps, the maximum MSD, through out the MD simulation, of the slow molecules was identified. The distribution of the maximum MSDs is shown in Figure S8b. Again, based on the initial peaks, it was assumed that any molecule with a maximum MSD equal to or below 10 nm^2, is considered trapped.

(a)

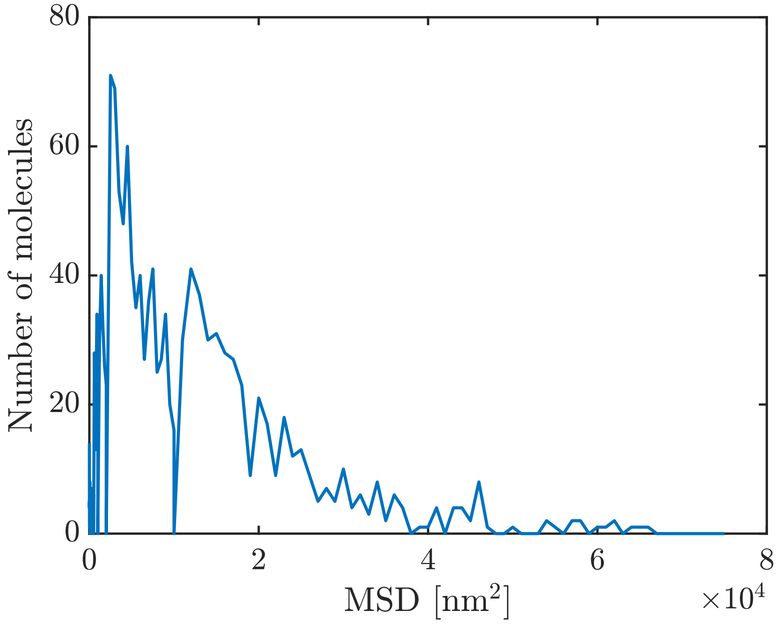

(b)

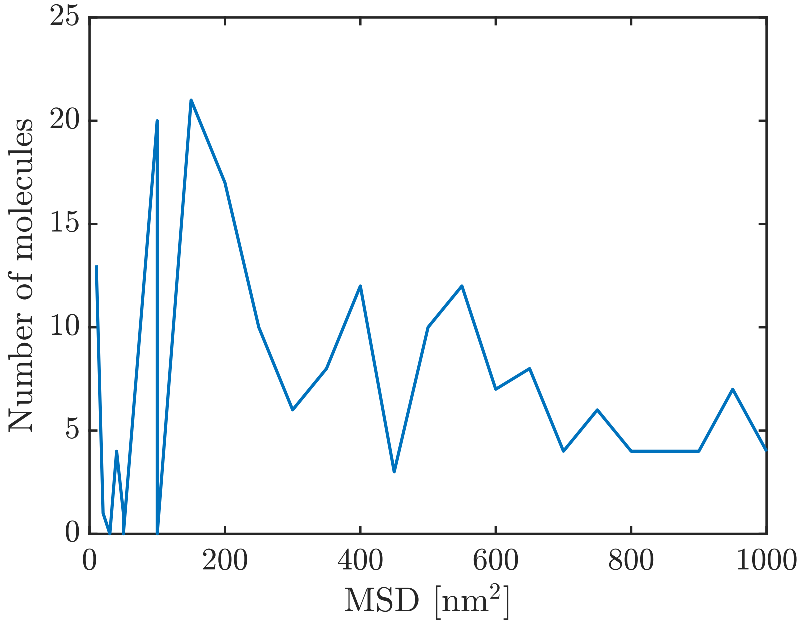

Figure S8: (a) Number of molecules per MSD value at the final time of the simulation (b) Number of molecules with maximum MSDs of slow molecules (with MSD of 1000 nm^2 or less at the final time). The analysis is reported for 1595 CH_4 molecules in pore 2. Details of MD simulations are provided in the main text.

5.2 Fitting MSDs

To find the lateral diffusion coefficient of CH_4 in a given region of the composite pore, the Fickian regime of the plot of MSD as function of time interval should be fitted. In Figure S9, MSD versus time intervals lines in the log scale are shown for each region. The regions are defined according to the density profile of CH_4 along the direction parallel to kaolinie walls. For each MSD curve, a line with a slope of 1 ($\log(MSD) = \log(t) + b$) is placed so that it tangent to MSD curve and identifying the Fickian regime where the relation $MSD_{xy} = 4D_{xy}t$ holds. The lateral diffusion coefficient can be directly estimated from the y-intercept of the line as $D_{xy} = \exp b/4$. For the cases where the a Fickian diffusion regime can not being identified (i.e. there is no region of the $\log(MSD)(\log(t))$ plot where the slope is one), indicative values of D_{xy} is found from fitting the MSD at the mean residence time as in the case of region 1.

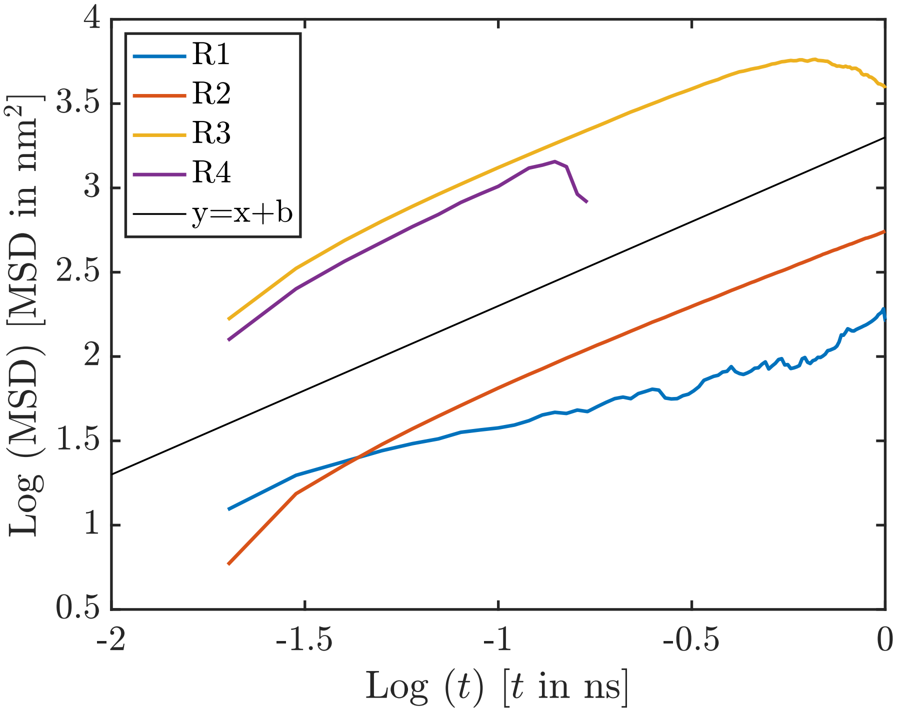

Figure S9: MSD versus time plots in the log scale of CH_4 in different regions of composite pore 2.

References

(1) Sarkisov, L.; Bueno-Perez, R.; Sutharson, M.; Fairen-Jimenez, D. Materials informatics with PoreBlazer v4.0 and the CSD MOF database. *Chemistry of Materials* **2020**, *32*, 9849–9867.

(2) Vasileiadis, M.; Peristeras, L. D.; Papavasileiou, K. D.; Economou, I. G. Modeling of bulk kerogen porosity: Methods for control and characterization. *Energy & Fuels* **2017**, *31*, 6004–6018.
